# HMGA2 Moderately Increases Fetal Hemoglobin Expression in Human Adult Erythroblasts

**DOI:** 10.1371/journal.pone.0166928

**Published:** 2016-11-18

**Authors:** Jaira F. de Vasconcellos, Y. Terry Lee, Colleen Byrnes, Laxminath Tumburu, Antoinette Rabel, Jeffery L. Miller

**Affiliations:** 1 Molecular Genomics and Therapeutics Section, Genetics of Development and Disease Branch, National Institute of Diabetes and Digestive and Kidney Diseases, National Institutes of Health, Bethesda, Maryland, United States of America; 2 Molecular Medicine Branch, National Institute of Diabetes and Digestive and Kidney Diseases, National Institutes of Health, Bethesda, Maryland, United States of America; University of Naples Federico II, ITALY

## Abstract

Induction of fetal hemoglobin (HbF) has therapeutic importance for patients with beta-hemoglobin disorders. Previous studies showed that *let-7* microRNAs (miRNAs) are highly regulated in erythroid cells during the fetal-to-adult developmental transition, and that targeting *let-7* mediated the up-regulation of HbF to greater than 30% of the total globin levels in human adult cultured erythroblasts. HMGA2 is a member of the high-mobility group A family of proteins and a validated target of the *let-7* family of miRNAs. Here we investigate whether expression of *HMGA2* directly regulates fetal hemoglobin in adult erythroblasts. *Let-7* resistant *HMGA2* expression was studied after lentiviral transduction of CD34(+) cells. The transgene was regulated by the erythroid-specific gene promoter region of the human *SPTA1* gene (HMGA2-OE). HMGA2-OE caused significant increases in *gamma-globin* mRNA expression and HbF to around 16% of the total hemoglobin levels compared to matched control transductions. Interestingly, no significant changes in *KLF1*, *SOX6*, *GATA1*, *ZBTB7A* and *BCL11A* mRNA levels were observed. Overall, our data suggest that expression of HMGA2, a downstream target of *let-7* miRNAs, causes moderately increased *gamma-globin* gene and protein expression in adult human erythroblasts.

## Introduction

Sickle cell disease (SCD) and the beta-thalassemias are among the most common genetic disorders worldwide [1, 2]. These diseases are caused by the occurrence of mutations in the adult globin gene [2–5]. Although many clinical trials are ongoing [6], the only drug currently approved by the Food and Drug Administration for the treatment of SCD is hydroxyurea, which is known to increase levels of fetal hemoglobin (HbF) [7]. HbF has a sparing effect in sickle hemoglobin polymerization [8], and HbF levels of >20% are sufficient to ameliorate the pathogenesis of the disease [9]. In recent years, many advances have been made in the investigation of factors that regulate HbF levels. Genome-Wide Association Studies (GWAS) identified several relevant polymorphisms to the variation of human HbF levels, including the *BCL11A* locus [10, 11]. Additional studies included the correction of the SCD phenotype by the knockdown of *BCL11A* in adult mice [12], and the discovery of an erythroid-specific enhancer that regulates the genetic variation of *BCL11A* levels [13, 14]. More recent studies identified the transcription factor *LRF*/*ZBTB7A* as causing robust increases in the levels of HbF in human cultured erythroblasts [15]. Altogether, these studies provide new insights into candidate-therapeutic targets for the treatment of beta-hemoglobin disorders; however a clinically effective approach for the reactivation of HbF in adult erythroblasts is still missing.

In erythroid cells, the *let-7* family of microRNAs (miRNAs) is highly regulated during the fetal-to-adult developmental transition, where *let-7* family members are consistently up-regulated in adult blood compared to cord blood reticulocytes [16]. Additional studies demonstrated that the *let-7* miRNAs, as well as LIN28A and LIN28B (proteins that bind and inhibit *let-7* miRNAs) regulate HbF expression levels to >30% of the total hemoglobin produced in cultured erythroblasts from adult humans [17]. Furthermore, over-expression of *LIN28A* was also shown to ameliorate the hypoxia-related sickling of cultured mature erythrocytes from pediatric patients with sickle cell disease [18]. More recently, high *LIN28B* expression was associated with elevated levels of HbF in patients with juvenile myelomonocytic leukemia, thus identifying expression of *LIN28B* as the hallmark of a novel fetal-like molecular subgroup in this disease [19].

High Mobility Group AT-hook 2 (*HMGA2*) is a member of the HMGA non-histone chromatin protein family known as ‘architectural transcription factors’ and are downstream targets of the *let-7* miRNAs. *HMGA2* mRNA is negatively-regulated by the binding of *let-7* miRNAs to the 3’-UTR region [20, 21]. Interestingly, *HMGA2* mRNAs are subjected to alternative splicing modifications and form four different isoforms that vary concerning their 3' structure, resulting in novel 3'-coding and -untranslated regions.

HMGA2 is known to both positively and negatively regulate the transcription of several genes by means of directly binding the DNA AT-hook domains (encoded by *HMGA2* exons 1 to 3) to the minor groove of AT-rich regulatory elements, altering the DNA conformation or the chromatin structure [22–24]. During development, HMGA2 has been shown to have a role in the regulation of growth and adipogenesis in mammalian animal models [24, 25]. Furthermore, HMGA2 is involved in a wide variety of biological mechanisms, such as cell-cycle progression, cell proliferation, cell differentiation and senescence [22, 26]. Of note, over-expression of *HMGA2* transcripts, possibly due to disruption of the *let-7* miRNAs binding sites at the *HMGA2* locus, accompanied an increased expression of endogenous HbF in a patient receiving lentiviral beta-globin gene therapy for the treatment of severe transfusion-dependent beta-thalassemia [27].

Here we investigated HMGA2 as a regulator of fetal hemoglobin. We observed moderate increases in *gamma-globin* gene expression and HbF levels in human adult cultured erythroblasts. Our data support that the LIN28-*let-7* heterochronic pathway increases HbF, in part, by the targeting of erythroblast *HMGA2* transcripts.

## Materials and Methods

### Ethics Statement

All related studies were performed after human subject review and Intramural National Institutes of Health Institutional Review Board approval. These studies were conducted in accordance with the Declaration of Helsinki. Written informed consent was obtained from all research subjects prior to participation in this study. Peripheral blood CD34(+) cells from healthy adult human volunteers were mobilized and isolated using a Clinimax device as previously described [28]. All cell processing, isolation, and cryopreservation were performed by personnel in the Cell Processing Section of the National Institutes of Health Department of Transfusion Medicine.

### Cell culture

Cryopreserved healthy adult human CD34(+) cells were cultured *ex vivo* in a serum-free system for 21 days consisting of three phases: phase I from days 0 to 7, phase II from days 7 to 14 and phase III from days 14 to 21, as previously described [17]. Cell counts were performed throughout the culture period in a Z1 Coulter Particle Counter (Beckman Coulter, Indianapolis, IN) following manufacturer’s instructions.

### Lentiviral erythroid promoter vector construction for HMGA2 over-expression

Lentiviral constructs were designed for *let-7* resistant expression of *HMGA2* variant 1 (HMGA2-OE) driven by the erythroid specific gene promoter region of the human *SPTA1* gene (GRCh38/hg381; chromosome 1: 158,686,528–158,687,488), with a matched empty vector control. *HMGA2* variant 1 was chosen because it is the only *HMGA2* variant with *let-7* binding sites in its 3’ UTR [20, 21]. Lentiviral backbone pLVX-IRES-Puro (Cat. 632183) was purchased from Clontech (Mountain View, CA). To generate the SPTA1-IRES-Puro plasmid (empty vector control), the CMV promoter from the pLVX-IRES-Puro vector was replaced with the human *SPTA1* promoter by directional cloning with ClaI and XhoI restriction enzymes as previously described [29]. The *HMGA2* coding region with added XhoI and BamHI restriction sites for directional cloning was amplified by PCR from human genomic DNA with the following PCR primer pairs: HG2A 5'XhoI: 5' ACCCTCGAGTATGAGCGCACGCGGTGAGGG 3'; V1HG2A 3'BamHI: 5' CCGGATCCCTAGTCCTCTTCGGCAGACTCTTGTGAGGAT 3' using CloneAmp HiFi PCR Premix (Clontech). The *HMGA2* PCR product was digested with XhoI and BamHI restriction enzymes (New England Biolabs, Ipswich, MA) following manufacturer’s protocol and cleaned up with MinElute Reaction Cleanup Kit (Qiagen, Valencia, CA), followed by cloning into the pLVX-SPTA1-IRES-Puro vector to generate a pLVX-SPTA1-HMGA2-IRES-Puro plasmid.

### HMGA2 virus production and lentiviral transduction

Virus production was performed as previously described [29]. Briefly, the plasmid mixture was prepared for co-transfection in HEK293T cells (Thermo Scientific, Waltham, MA) following the manufacturer’s instructions for the Calcium Phosphate Transfection Kit (Life Technologies, Grand Island, NY). The co-transfection mixture consisted of the vector plasmid (empty vector control or HMGA2-OE vector) with CAG kGP1.1R, CAG4 RTR2 and CAGGS vsv-g packaging helper virus plasmids [30, 31]. The lentivirus-containing supernatant was concentrated following the Lenti-X Concentrator (Clontech) manufacturer’s protocol and resuspended in 1/100 of the original supernatant volume in phase I culture medium. The presence of lentiviral particles was confirmed using the Lenti-X GoStix (Clontech) following the manufacturer’s instructions. Cryopreserved CD34(+) cells were thawed and seeded at a density of 250,000 cells/ml in phase I culture medium. On culture day 3, cells were resuspended at a density of 2,000 cells/μl in phase I culture medium and transduced with lentiviral particles (an estimated MOI of 5 was calculated for the viral transductions). On the day after transduction, cells were resuspended in 4.0 ml phase I culture medium containing puromycin and transferred on day 7 into phase II culture medium without puromycin. For each condition, a puromycin selected control of mock-transduced cells was cultured in matched conditions until the end of phase II and analyzed to confirm puromycin selection by flow cytometry.

### Flow cytometry analyses

Erythroid differentiation was assessed with transferrin receptor (CD71) and glycophorin A (GPA) antibodies (Invitrogen, Carlsbad, CA) on culture days 14 and 21 using the BD FACSAria I flow cytometer (BD Biosciences, San Jose, CA) as previously described [32]. Enucleation was quantitated by thiazole orange staining (Sigma) on culture day 21. To assess fetal hemoglobin, cells were stained with antibody directed against HbF (Invitrogen).

### Quantitative PCRs

Total RNA was isolated using miRNeasy mini kit with QIAzol following manufacturer’s instructions (Qiagen) and quantitated by reading absorbance at 260 nm in the spectrophotometer. In all samples, complementary DNA (cDNA) was synthesized using SuperScript III reverse transcriptase (Thermo Fisher) from the same amount of total RNA following manufacturer’s instructions and as previously described [28]. Quantitative real-time PCR amplification was carried out in a 7700 Sequence Detection System using TaqMan Universal PCR Master Mix (Thermo Fisher) used following manufacturer’s instructions. Q-RT-PCR assays and conditions were performed as previously described [17, 33, 34], with the addition of *ZBTB7A* (Hs00792219_m1) Assay-on-Demand Gene Expression Product (Thermo Fisher/Applied Biosystems, Grand Island, NY) for gene expression analyses. For the *let-7* family of miRNAs, cDNA synthesis and Q-RT-PCR assays and conditions were performed as previously described [16] for *let-7a*, *let-7b*, *let-7c*, *let-7d*, *let-7e*, *let-7f*, *let-7g*, *let-7i* and *miR-98*. In all Q-RT-PCR reactions, absolute quantification for each transcript was determined by comparison with a standard curve that was run in parallel with biological samples as previously described [28]. For each *let-7* family member, standard curves were constructed on the basis of the synthetic targeted mature miRNA oligonucleotide of known concentration (1:10 serial dilutions, n = 6). Reactions were performed in triplicate. A file containing each of the standard curves statistics is included as S1 Appendix.

### Western blot analysis

Nuclear and cytoplasmic extracts were prepared using the NE-PER Nuclear and Cytoplasmic Extraction kit (Pierce Biotechnology, Rockford, IL) from culture day 14 erythroblasts as previously described [17]. Equal amounts of protein were separated following the Western blot protocol as previously described [17]. Blots were probed with antibody against HMGA2 (Abcam, Cambridge, MA). Lamin B1 was used as loading control (Abcam, Cambridge, MA).

### Confocal analysis

For confocal microscopy, slides containing 70,000 sorted live cells on culture day 14 were fixed with 3.7% paraformaldehyde in PBS (Electron Microscopy Sciences, Hatfield, PA) and permeabilized with 2% BSA/PBS containing 0.1% Triton X (Thermo Fisher Scientific, Grand Island, NY). After cell permeabilization, slides were probed with antibody against human HMGA2 (1:500, ab184616, Abcam) overnight at 4°C in 2% BSA/PBS solution. Slides were incubated with secondary antibody against rabbit IgG (H+L) conjugated with CF-555 (1:500, Sigma) in 2% BSA/PBS solution for one hour at room temperature. After secondary antibody incubation, slides were washed three times in PBS and once in distilled water then mounted with ProLong Diamond Antifade Mountant with DAPI (Thermo Fisher Scientific). Laser scanned confocal images were obtained from LSM 5 Live Duoscan (Carl Zeiss, Oberkochen, Germany) and analyzed with Zen2007 software.

### HPLC for adult and fetal hemoglobins

HPLC samples were prepared and analyzed as previously described [28, 35].

### Statistical analysis

Replicates are expressed as mean ± SD values and significance was calculated by one- or two-tailed paired Student’s t-test.

## Results

### HMGA2 over-expression has minor effects in erythroblast *in vitro* differentiation and enucleation

**Fig 1:**
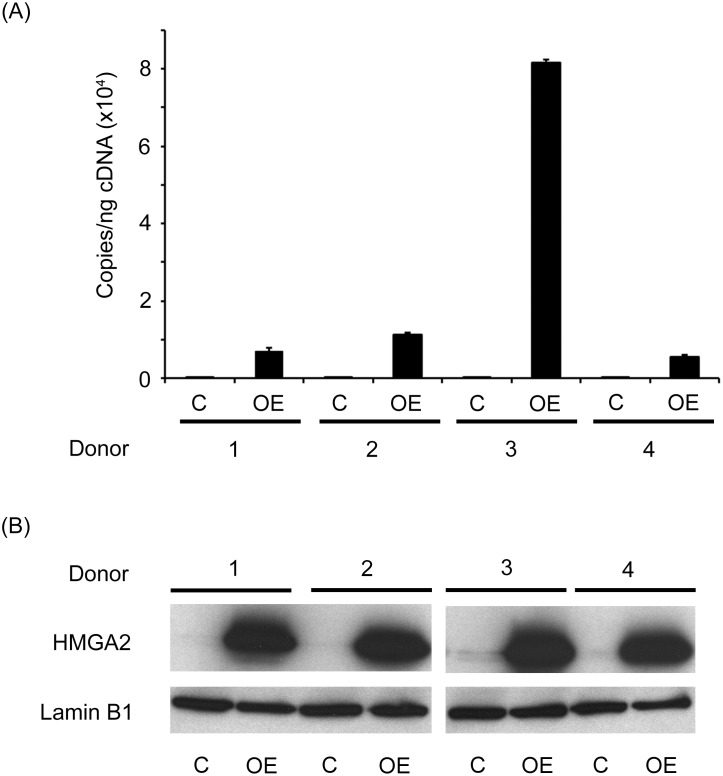
HMGA2 over-expression was confirmed at the mRNA and protein levels. **(A)** Quantitation of copy number per nanogram cDNA (copies/ng cDNA) by Q-RT-PCR in empty vector control and HMGA2-OE samples. Open bars represent empty vector control and black bars represent HMGA2-OE. Four independent donors were performed for each condition. **(B)** Western blot analysis of HMGA2 in the nuclear extracts from four independent donors upon HMGA2-OE compared to transduction controls. Blot was probed with anti-HMGA2 antibody and Lamin B1 was used as loading control. C = empty vector control transduction; OE = HMGA2 over-expression.

HbF regulation in humans remains an active area of investigation. Recently, the heterochronic LIN28-*let-7* pathway that has been associated with developmental timing events in *C*. *elegans* has been reported to be involved in the modulation of HbF levels [17, 19]. Since the LIN28 RNA-binding proteins negatively regulate the *let-7* family of miRNAs [36] and the *let-7* miRNAs are negative regulators of *HMGA2* [21], we investigated the role of HMGA2 in the regulation of HbF. For this purpose, erythroid-specific over-expression of *HMGA2* was performed and compared to empty vector controls. To prevent degradation in the adult CD34(+) cells, the 3’-untranslated region (UTR) of the gene where the *let-7* miRNAs binding sites are located was excluded, and only the open reading frame from the *HMGA2* variant 1 was used in the over-expression lentivirus transgene, keeping intact the region encoding the DNA-binding AT-hook domains. Transductions were performed in CD34(+) cells from adult healthy volunteers cultivated *ex vivo* in erythropoietin-supplemented serum-free media for 21 days. Expression of transgenic HMGA2 (HMGA2-OE) was confirmed by Q-RT-PCR (empty vector control: below detection limits; HMGA2-OE: 2.51E+04 ± 3.44E+04 copies/ng) and Western analyses at culture day 14 (Fig 1). Pancellular HMGA2 expression was demonstrated by confocal analysis of HMGA2-OE compared to control transduction (S1 Fig).

**Fig 2:**
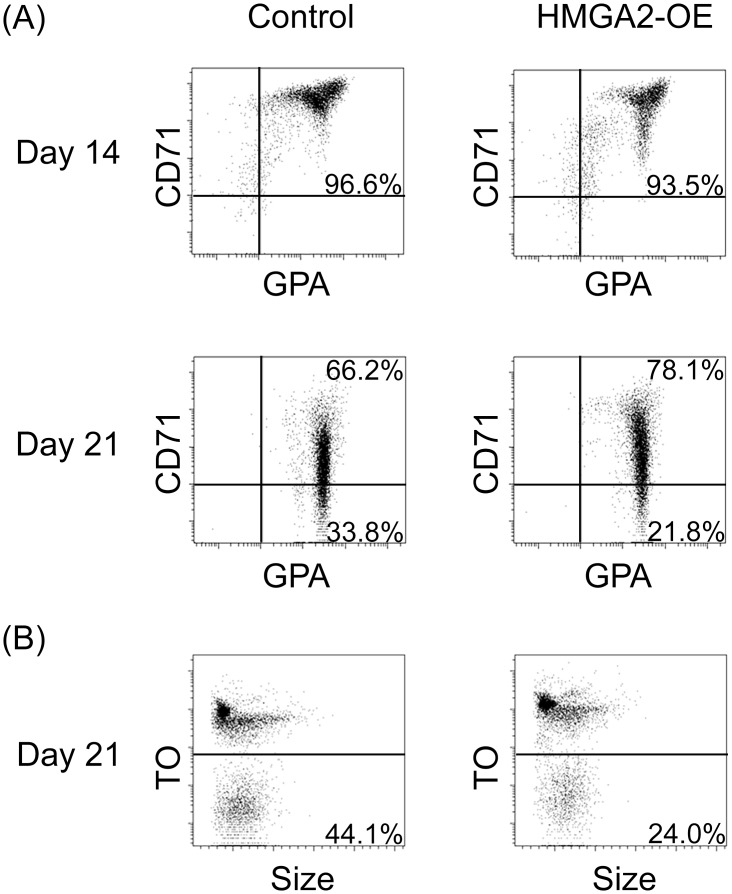
Erythroid-expression of HMGA2 leads to slightly lower levels of terminal maturation and enucleation compared to control transductions during *in vitro* erythroblast differentiation. Representative donor flow cytometry analyses of **(A)** empty vector control and HMGA2-OE at culture days 14 and 21 stained with anti-transferrin receptor (CD71) and anti-glycophorin A (GPA) antibodies. **(B)** Enucleation of empty vector control and HMGA2-OE transductions assessed by staining of culture day 21 cells with thiazole orange. Percentages shown in the figure correspond to one representative donor, average values are shown in the text. C = empty vector control transduction; HMGA2-OE = HMGA2 over-expression.

Cell proliferation and erythroblast differentiation were compared between empty vector control samples and HMGA2-OE. Cell counts at culture day 14 showed no significant differences between HMGA2-OE and empty vector control transductions (S2 Fig). Furthermore, erythroblast maturation characterized by expression and gradual loss of transferrin receptor (CD71) and expression of glycophorin A (GPA) on the cell surface was analyzed in the empty vector control and HMGA2-OE samples at day 14 and at the end of the culture period (Fig 2A), as well as enucleation was assessed by thiazole orange (TO) staining in the empty vector control and HMGA2-OE samples at the end of the culture period (Fig 2B). Maturation was equivalent among HMGA2-OE and empty vector control cells at culture day 14. However, less terminal maturation and enucleation were detected by culture day 21 [Average % CD71(+) and GPA(+): empty vector control: 54.4 ± 9.7, HMGA2-OE: 71.6 ± 11.1, p = 0.01; Average % CD71(-) and GPA(+): empty vector control: 45.6 ± 9.7, HMGA2-OE: 28.3 ± 11.1, p = 0.01]. Despite the decreased maturation, HMGA2-OE cells maintained the ability to enucleate to an average of 35.2 ± 17.0% in the *ex vivo* culture conditions.

### HMGA2 over-expression moderately regulates HbF levels in human adult erythroblasts

**Fig 3:**
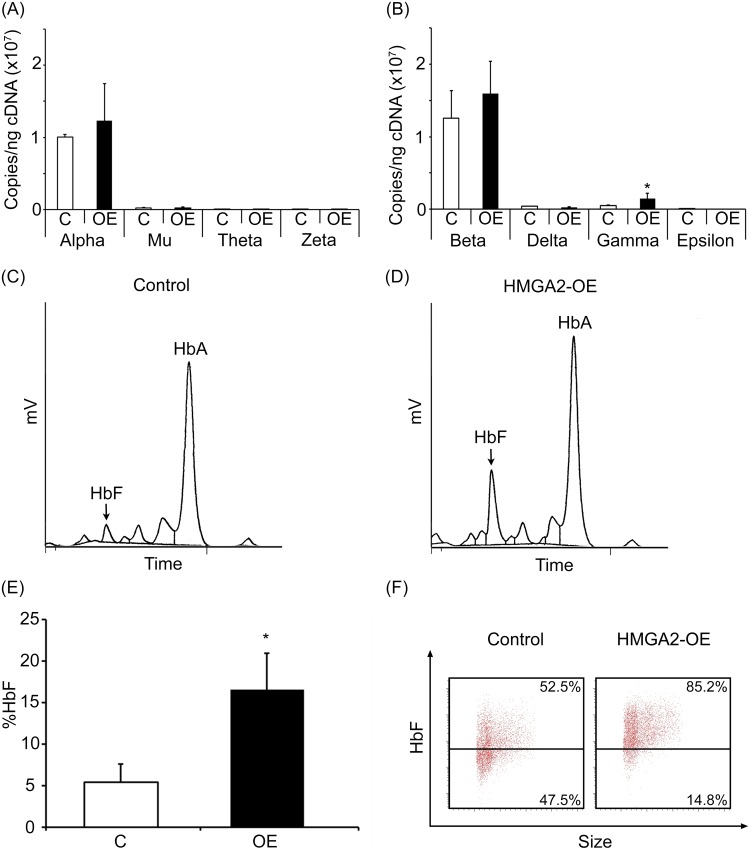
Enforced expression of HMGA2 regulates *gamma-globin* mRNA and pancellular HbF levels in adult human erythroblasts cultivated *ex vivo*. Quantitation of copy number per nanogram cDNA (copies/ng cDNA) by Q-RT-PCR analyses was performed at culture day 14 for **(A)** *alpha-*, *mu-*, *theta-* and *zeta-globins*, and **(B)** *beta-*, *delta-*, *gamma-* and *epsilon-globins* in HMGA2-OE and empty vector control transductions. Representative HPLC profile of hemoglobin collected at culture day 21 from **(C)** empty vector control transductions and **(D)** HMGA2-OE. **(E)** Average percentage of HbF levels detected by HPLC analysis. **(F)** Representative flow cytometry dot plots of empty vector control transductions (left panel) and HMGA2-OE (right panel) at culture day 21 stained for fetal hemoglobin. Open bar represents empty vector control and black bar represents HMGA2-OE. Mean value ± SD of four independent donors for each condition. P-value was calculated using one-tailed paired Student’s t-test. C = empty vector control transduction; OE = HMGA2 over-expression. *p<0.05.

To investigate the effects of HMGA2-OE in the expression levels of the globin mRNAs in cultured adult erythroblasts, Q-RT-PCR analysis of the *alpha*- and *beta-globin* genes was performed at culture day 14. No major differences were observed in *mu*-, *theta*-, *zeta*-, *delta*- and *epsilon*-*globin* transcripts in HMGA2-OE samples compared to empty vector control transductions (Fig 3A and 3B). Average increases of 23% and 27% in mRNA levels of *alpha*- and *beta*-*globin* were detected, respectively, but those increases did not reach statistical significance due to donor variability (S3A and S3B Fig). In contrast, *gamma-globin* mRNA was consistently increased in each donor resulting in an average increase of 189% observed in HMGA2-OE compared to controls (empty vector control: 5.02E+05 ± 8.62E+04 copies/ng; HMGA2-OE: 1.45E+06 ± 7.31E+05 copies/ng; p = 0.037; Fig 3B and S3C Fig). HPLC analyses at culture day 21 demonstrated a modest but significant increase in HbF levels in HMGA2-OE compared to controls (empty vector control: 5.41 ± 2.15%; HMGA2-OE: 16.53 ± 4.43%; p = 0.003; Fig 3C–3E and S3D Fig), which is consistent with the increase in *gamma-globin* mRNA levels. In addition, HbF staining by flow cytometry at culture day 21 demonstrated that HMGA2-OE has a pancellular expression of HbF compared to the control transductions (Fig 3F). Overall, the data showed here supports the notion that the LIN28-*let-7* pathway regulates HbF, at least in part, by targeting of the erythroblast *HMGA2* transcripts.

### HMGA2 over-expression has minor effects in erythroid- and HbF-related transcription factors

**Fig 4:**
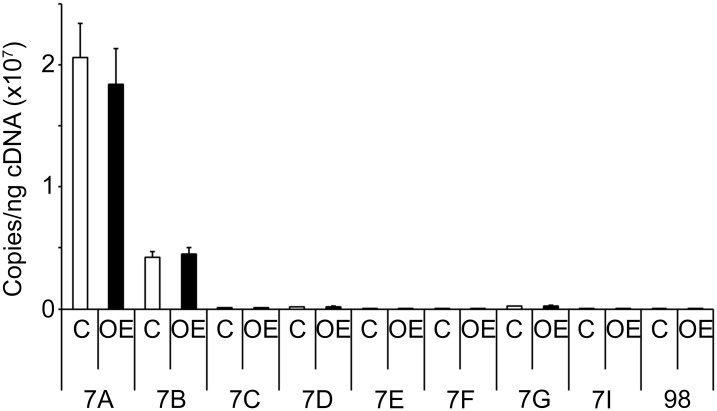
HMGA2 does not regulate the *let-7* family of miRNAs. Effects in the expression levels of the *let-7* family of miRNAs were analyzed in HMGA2-OE and empty vector control transductions. Quantitation of copy number per nanogram cDNA (copies/ng cDNA) by Q-RT-PCR analyses was performed at culture day 14. Open bars represent empty vector control and black bars represent HMGA2-OE. Mean value ± SD of four independent donors for each condition. C = empty vector control transduction; OE = HMGA2 over-expression.

**Fig 5:**
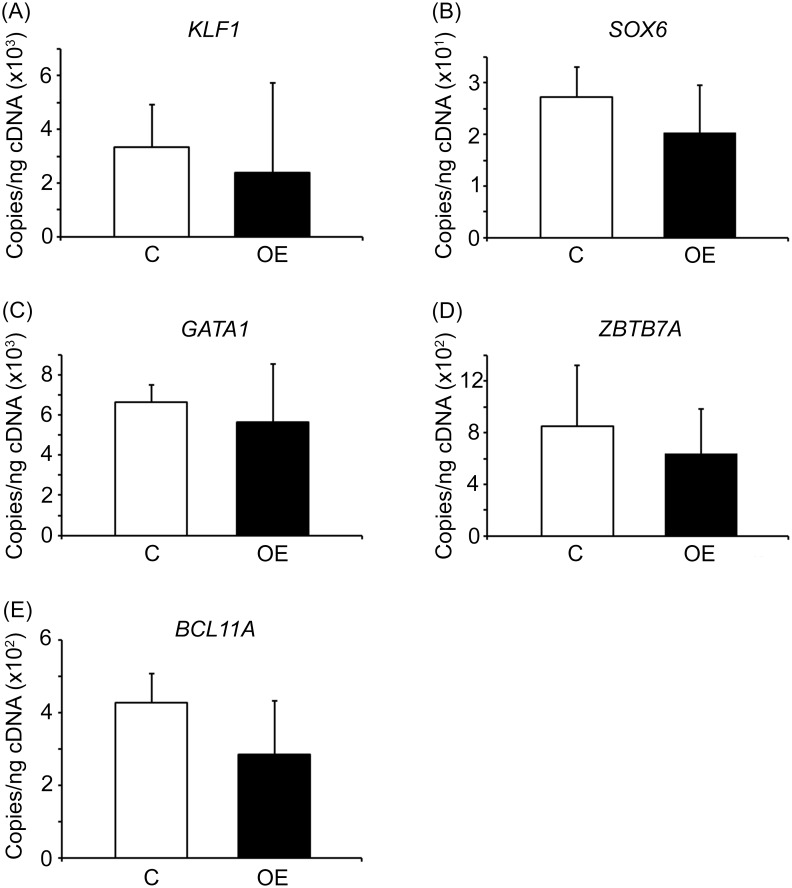
HMGA2 expression has mild effects in erythroid- and HbF-related transcription factors. Expression level of **(A)** *KLF1*, **(B)** *SOX6*, **(C)** *GATA1*, **(D)** *ZBTB7A* and **(E)** *BCL11A* were analyzed in HMGA2-OE and empty vector control transductions. Quantitation of copy number per nanogram cDNA (copies/ng cDNA) by Q-RT-PCR analyses was performed at culture day 14. Open bars represent empty vector control and black bars represent HMGA2-OE. Mean value ± SD of four independent donors for each condition. C = empty vector control transduction; OE = HMGA2 over-expression.

To determine if HMGA2-OE acts via known regulators of *gamma-globin*, Q-RT-PCR analyses were performed for expression of the *LIN28* genes, the *let-7* family of miRNAs as well as several erythroid-related transcription factors. *LIN28A* and *LIN28B* were not affected by HMGA2-OE remaining below the detection limits. Likewise, no significant changes were detected in the levels of the *let-7* family of miRNAs in HMGA2-OE compared to empty vector control samples (Fig 4). These results demonstrate that upon high-level erythroblast expression of HMGA2 there is no feedback loop that regulates *LIN28* transcripts or the *let-7* miRNAs. In addition, the expression pattern of the transcription factors *KLF1*, *SOX6*, *GATA1*, *ZBTB7A* and *BCL11A* were investigated and, despite a down-regulation trend in the expression level of these transcription factors, there were no statistically significant changes detected in HMGA2-OE compared to empty vector control transductions (Fig 5A–5E). Overall, HMGA2-OE caused mild changes in several transcription factors that are known regulators of HbF, but none of the changes reached statistical significance in this study.

## Discussion

Understanding the molecular processes controlling the fetal-to-adult hemoglobin transition is crucial to determine the drivers and effectors of HbF, which have the potential to be used in the treatment of beta-hemoglobin disorders. Here we focus on recent discoveries that highlight the interplay between the heterochronic LIN28-*let-7* pathway and the regulation of HbF. Our results demonstrate that HMGA2, a downstream target of the *let-7* miRNAs [37], plays a moderate role in the modulation of *gamma-globin* and HbF levels. Importantly, HMGA2 had only modest effects in the activation of HbF, so these results are unable to fully explain the more robust HbF effects of *let-7* miRNA suppression by LIN28 in the same culture model [17].

*HMGA2* is physiologically expressed during embryonic and fetal developmental stages, and it is down-regulated to very low or absent levels in normal adult tissues [38]. In addition, *Hmga2* transcripts are differentially expressed between fetal and adult mice hematopoietic stem cells [39]. Here we expressed a *let-7*-resistant HMGA2 variant 1 driven by the erythroid specific gene promoter region of the human *SPTA1* gene. Of note, HMGA2 proteins do not have a direct transcriptional activation function; however, they can control gene expression by interaction with DNA and transcription factors (TFs) to form complexes that support the TFs with binding to the DNA [40], or exclusively interacting with the TFs and, consequently, increasing their affinity to bind to its target DNA [41].

It has been shown that transgenic mice carrying a 3'UTR-truncated *Hmga2* cDNA showed proliferative hematopoiesis, erythropoietin-independent erythroid colony formation, as well as hypercellular bone marrow and splenomegaly [42]. Conversely, similar hyperproliferative changes were not associated with increased expression of a truncated HMGA2 protein, accompanied by an increase in expression of endogenous HbF, in a patient receiving lentiviral beta-globin gene therapy for the treatment of beta-thalassemia [27]. We report here that erythroid-specific-*let-7*-resistant HMGA2 over-expression had only minor effects in *ex vivo* erythropoiesis and enucleation. While mRNA levels of *alpha*- and *beta-globin* genes were both increased upon HMGA2 over-expression, these increases were most likely the result of donor variability (S3 Fig). However, a moderate and consistent activation of *gamma-globin* expression was observed upon HMGA2 over-expression in all donors, as well as increased and pancellular HbF levels to an average of 16% of the total hemoglobin produced. Despite the high HMGA2 protein levels detected in each donor’s cells, HbF was increased to levels lower than those observed in previous studies directly targeting *LIN28* or *let-7* miRNAs performed under similar culture conditions [17]. Due to a modest effect on HbF with HMGA2-OE, additional factors are being sought that may act in combination with HMGA2 for the activation of HbF in adult erythroblasts, such as other downstream mRNA targets of the *let-7* family of miRNAs.

Over-expression of HMGA2 did not significantly affect the levels of *LIN28* or any of the *let-7* family members, suggesting that the effects were not mediated by feedback regulation within the *let-7* regulatory circuit. To explore potential downstream effects of HMGA2 over-expression in adult erythroblasts, the expression levels of transcription factor regulators of HbF were investigated. *KLF1*, *SOX6*, *GATA1*, *ZBTB7A* and *BCL11A* mRNA levels were not significantly reduced with the over-expression of HMGA2. We interpreted these results as an indication that HMGA2 effects are not primarily mediated by a singular transcription factor in this group. Alternatively, the subtle differences in the erythroblast differentiation (Fig 2) mediated by HMGA2-OE may influence the effects of transcription factors upon globin gene regulation as well as HbF levels.

Finally, our studies on HMGA2 add to recent efforts aimed toward developing an experimental bridge that connects the ancient and well-conserved LIN28-*let-7* developmental timing circuit with the abundant knowledge of globin gene regulation during human ontogeny. Importantly, since direct targeting of the *let-7* miRNAs in adult erythroblasts causes more robust effects in the reactivation of HbF levels, the results shown here suggest that HMGA2 expression is not sufficient to fully explain the downstream effects of the LIN28-*let-7* circuit in the regulation of HbF levels.

## Supporting Information

S1 Appendix Quantitative PCRs standard curves statistics. (XLSX)

S1 Fig HMGA2 over-expression has a pancellular distribution in adult human erythroblasts cultivated *ex vivo*. Confocal analysis of empty vector control transduction (control) and HMGA2 over-expression (HMGA2-OE) were performed at culture day 14. Confocal images of control transduction and HMGA2 over-expression cells were stained with DAPI (4’,6-diamidino-2-phenylindole) (blue) and HMGA2 (green).
(TIF)

S2 Fig Erythroid-expression of HMGA2 demonstrates comparable levels of cell proliferation compared to control transductions. Cell proliferation was assessed by cell counts (cells/mL) performed at culture day 14. Open bar represents empty vector control and black bar represents HMGA2-OE. Mean value ± SD of four independent donors for each condition. C = empty vector control transduction; OE = HMGA2 over-expression.
(TIF)

S3 Fig Effects of *HMGA2* over-expression on the levels of **(A)** *alpha*-, **(B)** *beta*-, and **(C)** *gamma-globin* transcripts according to individual donors. Quantitation of copy number per nanogram cDNA (copies/ng cDNA) was performed by Q-RT-PCR at culture day 14. Standard deviation was calculated for each sample in triplicate reactions. **(D)** Effects of *HMGA2* over-expression on the levels of fetal hemoglobin (values for each donor are shown). Fetal hemoglobin levels were measured by HPLC analysis of hemoglobin collected at culture day 21 from HMGA2-OE and empty vector control transductions. Open bars represent empty vector control and black bars represent the HMGA2 over-expression. C = empty vector control transduction; OE = HMGA2 over-expression.
(TIF)
