# Supplementary figures and images for: HMGA2 Moderately Increases Fetal Hemoglobin Expression in Human Adult Erythroblasts
Source: PLoS One. 2016 Nov 18;11(11):e0166928. doi: 10.1371/journal.pone.0166928 (PMC5115839; doi:10.1371/journal.pone.0166928)

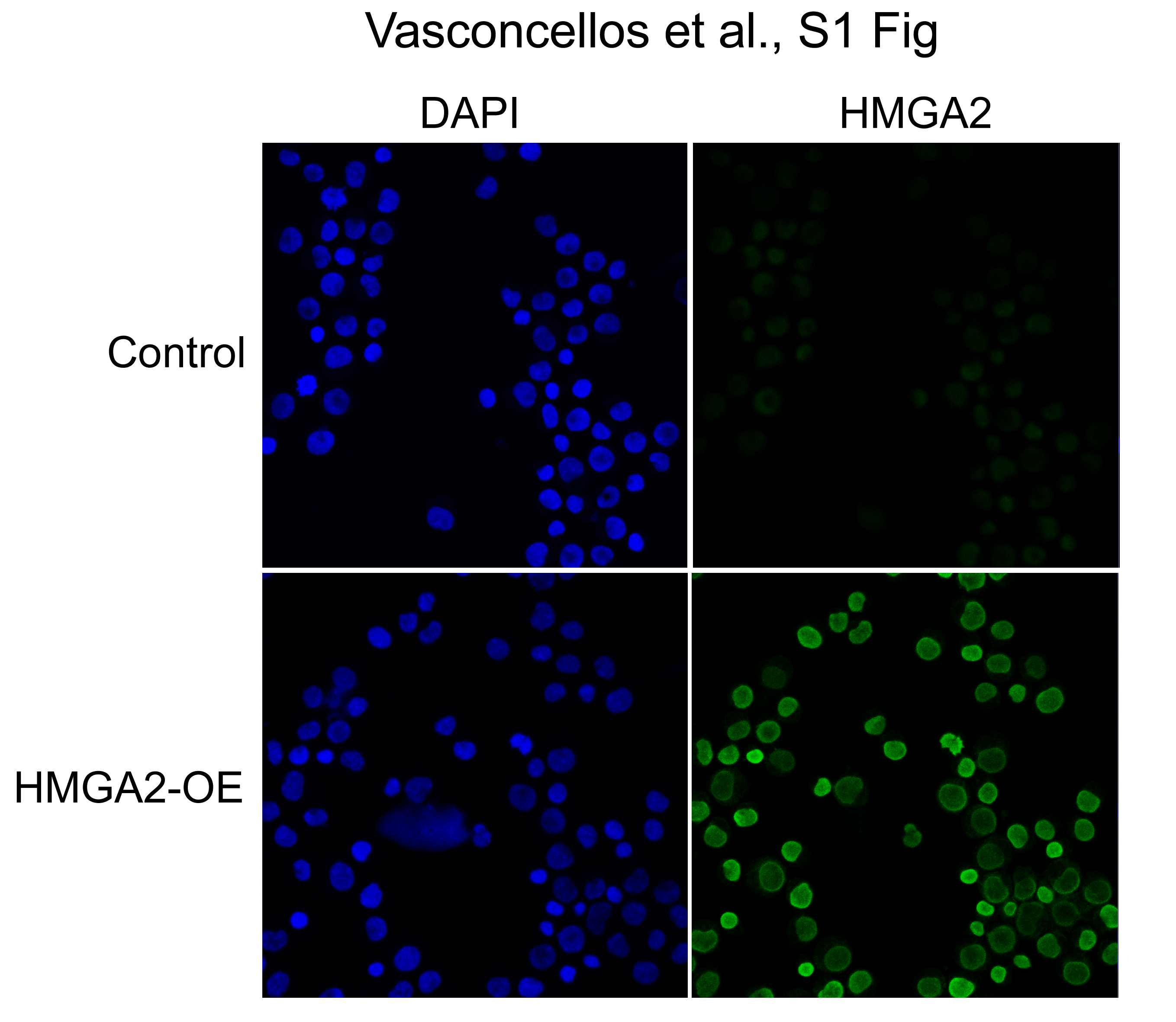

Supplement: S1 Fig — Confocal analysis of empty vector control transduction (control) and HMGA2 over-expression (HMGA2-OE) were performed at culture day 14. Confocal images of control transduction and HMGA2 over-expression cells were stained with DAPI (4’,6-diamidino-2-phenylindole) (blue) and HMGA2 (green). (TIF) [file pone.0166928.s002.tif]

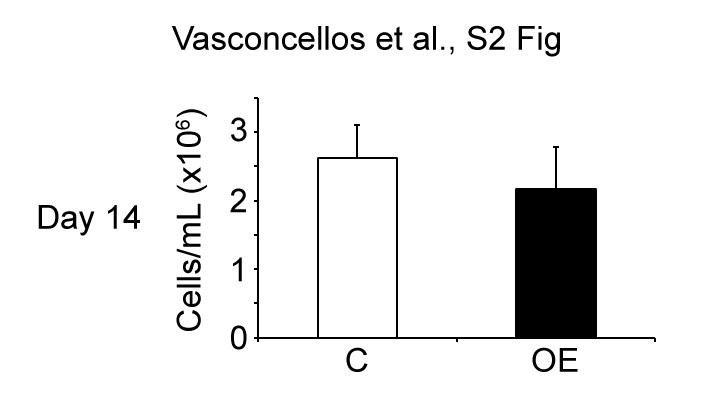

Supplement: S2 Fig — Cell proliferation was assessed by cell counts (cells/mL) performed at culture day 14. Open bar represents empty vector control and black bar represents HMGA2-OE. Mean value ± SD of four independent donors for each condition. C = empty vector control transduction; OE = HMGA2 over-expression. (TIF) [file pone.0166928.s003.tif]

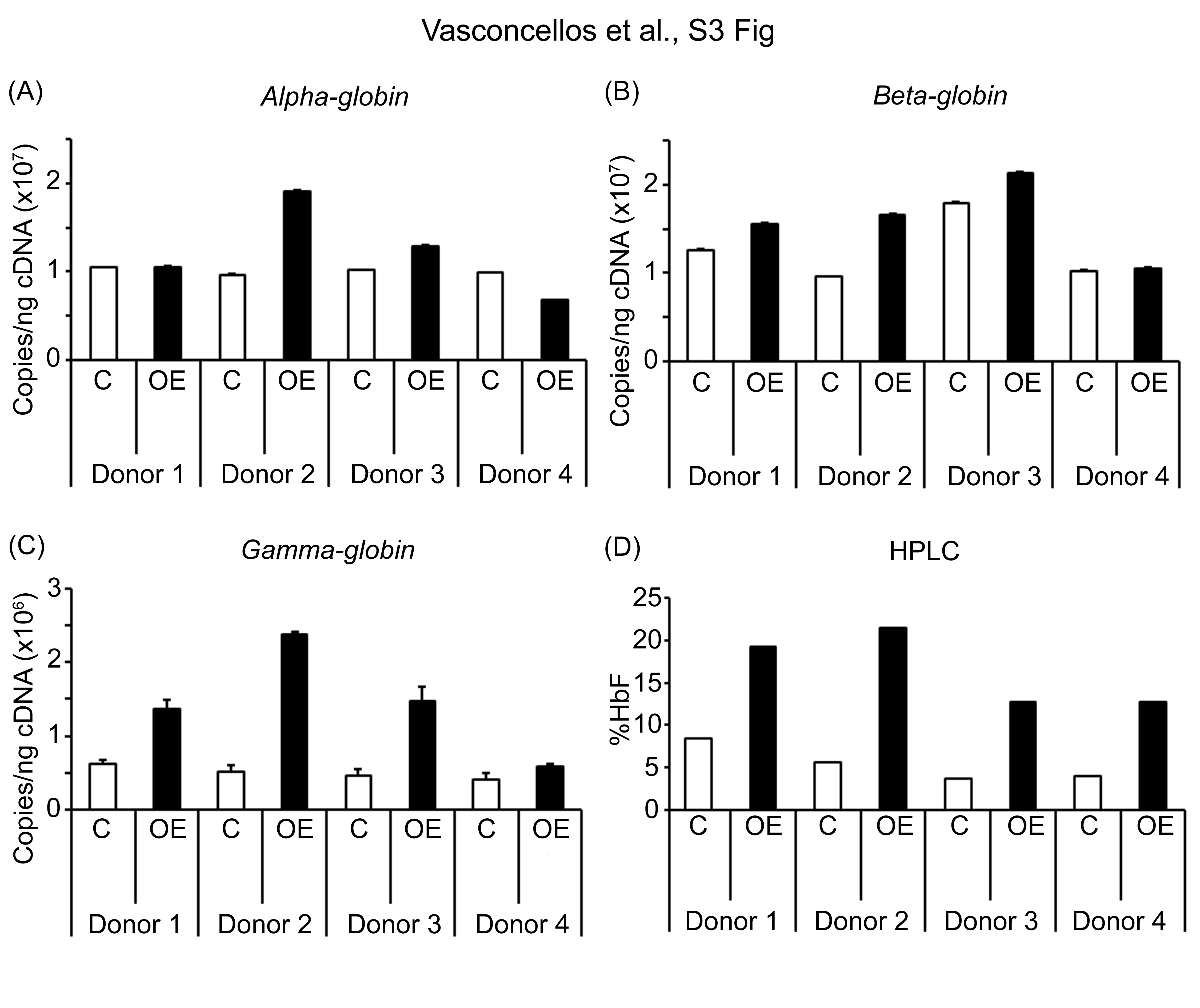

Supplement: S3 Fig — Effects of HMGA2 over-expression on the levels of (A) alpha-, (B) beta-, and (C) gamma-globin transcripts according to individual donors. Quantitation of copy number per nanogram cDNA (copies/ng cDNA) was performed by Q-RT-PCR at culture day 14. Standard deviation was calculated for each sample in triplicate reactions. (D) Effects of HMGA2 over-expression on the levels of fetal hemoglobin (values for each donor are shown). Fetal hemoglobin levels were measured by HPLC analysis of hemoglobin collected at culture day 21 from HMGA2-OE and empty vector control transductions. Open bars represent empty vector control and black bars represent the HMGA2 over-expression. C = empty vector control transduction; OE = HMGA2 over-expression. (TIF) [file pone.0166928.s004.tif]
